# Circular RNA circHIPK3 Promotes the Proliferation and Differentiation of Chicken Myoblast Cells by Sponging miR-30a-3p

**DOI:** 10.3390/cells8020177

**Published:** 2019-02-19

**Authors:** Biao Chen, Jiao Yu, Lijin Guo, Mary Shannon Byers, Zhijun Wang, Xiaolan Chen, Haiping Xu, Qinghua Nie

**Affiliations:** 1 Department of Animal Genetics, Breeding and Reproduction, College of Animal Science, South China Agricultural University, Guangzhou 510642, China; biaochen@stu.scau.edu.cn (B.C.); 13539763630@163.com (J.Y.); guolijin2016@163.com (L.G.); zhijunwang@stu.scau.edu.cn (Z.W.); xiaolanchen@stu.scau.edu.cn (X.C.); music-three@163.com (H.X.); 2 National-Local Joint Engineering Research Center for Livestock Breeding, Guangzhou 510642, China; 3 Guangdong Provincial Key Lab of Agro-Animal Genomics and Molecular Breeding, and Key Laboratory of Chicken Genetics, Breeding and Reproduction, Ministry of Agriculture, Guangzhou 510642, China; 4 Department of Biological Sciences, College of Life and Physical Sciences, Tennessee State University, Nashville, TN 37209, USA; maryshannonbyers@yahoo.com

**Keywords:** circular RNA, circHIPK3, microRNA, miR-30a-3p, skeletal muscle, proliferation, differentiation

## Abstract

Circular RNAs and microRNAs widely exist in various species and play crucial roles in multiple biological processes. It is essential to study their roles in myogenesis. In our previous sequencing data, both miR-30a-3p and circular HIPK3 (circHIPK3) RNA, which are produced by the third exon of the *HIPK3* gene, were differentially expressed among chicken skeletal muscles at 11 embryo age (E11), 16 embryo age (E16), and 1-day post-hatch (P1). Here, we investigated their potential roles in myogenesis. Proliferation experiment showed that miR-30a-3p could inhibit the proliferation of myoblast. Through dual-luciferase assay and Myosin heavy chain (MYHC) immunofluorescence, we found that miR-30a-3p could inhibit the differentiation of myoblast by binding to Myocyte Enhancer Factor 2 C (*MEF2C*), which could promote the differentiation of myoblast. Then, we found that circHIPK3 could act as a sponge of miR-30a-3p and exerted a counteractive effect of miR-30a-3p by promoting the proliferation and differentiation of myoblasts. Taking together, our data suggested that circHIPK3 could promote the chicken embryonic skeletal muscle development by sponging miR-30a-3p.

## 1. Introduction

Skeletal muscles are important components in animals. Chicken skeletal muscle, which can provide high quality protein, is one of the most important meat source for humans. The development of skeletal muscle is regulated by multiple factors, including genetics, nutrition, disease, environment and so on [1,2]. Heritability estimates showed that chicken growth could be enhanced by genetic improvement [3]. The genetic factors which control skeletal muscle development include genes and non-coding RNAs.

MicroRNAs (miRNAs) have been shown to be involved in many biological processes, including muscle development [4]. Some myogenic miRNAs, including the miR-1 family, miR-206 and miR-133 family, regulate muscle development by targeting and inhibiting the expression of muscle-related gene [5,6]. Previous studies in our group showed that miR-203, miR-16, miR-29, and miR-1611 all played crucial roles in myoblast proliferation and differentiation [7,8,9,10]. Two other studies found that miR-30a-3p could suppress tumor growth [11,12]. However, the molecular function of chicken miR-30a-3p has not yet been reported.

Circular RNAs, which widely exist in the transcriptomes of different species and tissues, were previously considered as a kind of non-coding RNA, but they have now been demonstrated to have both coding and regulating functions [13,14,15]. Circular RNA, formed by the covalently joined 5′ and 3′ ends of linear RNA, possess a more stable structure than linear RNA. The functions of circular RNA include, acting as a miRNA sponge, participating in regulating the expression of its own linear RNA in different ways, coding protein, and deriving pseudogenes [16,17]. Previous studies found that circular RNAs were abundantly expressed in skeletal muscle tissue in many species [14,18,19]. Circular RNAs in chicken skeletal muscle could act as an miRNA sponge and regulate chicken muscle development [19]. A circular RNA, produced by *SVIL* could promote the proliferation and differentiation of myoblast cells by sponging miR-203 [20]. Circular RNA circFGFR2, generated by the *FGFR2* gene, could interact with miR-133a-5p and miR-29b-1-5p to regulate myoblast cells development [7].

A circular RNA produced by the third exon of the chicken *HIPK3* gene (circHIPK3—01, we named it as circHIPK3, hereinafter) has the highest expression level compare to other circular RNAs generated from *HIPK3* gene. It was also differentially expressed in different stages of skeletal development. We predicted it has three potential binding sites for miR-30a-3p. In this study, we aimed to examine the interaction of circHIPK3 and miR-30a-3p and their functions on myoblast proliferation and differentiation.

## 2. Materials and Methods

### 2.1. Ethics Statement

All animal experiments performed in this study met the requirements of the Institutional Animal Care and Use Committee at the South China Agricultural University (approval ID: SCAU#0014). All efforts were made to minimize the suffering of animals.

### 2.2. Primers

All primers that were used in this study were synthesized by Sangon (Sangon Biotech, Shanghai, China). The primers listed in Table 1 were designed by Premier Primer 5.0 software (Premier Bio-soft International, Palo Alto, CA, USA). Information of the qRT-PCR primers for *MYOD*, *MYOG*, *MYHC* and *GAPDH* were shown in our previous study [21].

### 2.3. Cell Culture and Transfection

Chicken primary myoblasts (CPMs) were isolated from the leg muscle of 10-day Yuhe chicken embryos (E10; Zhuhai Yuhe Company Ltd., Zhuhai, China), as described in our previous study [7]. Briefly, the legs of E10 chickens were collected, and the skin and bones were removed. Then, leg muscles were minced with scissors and trypsinized (Gibco, Grand Island, NY, USA) at 37 °C for 20 min). Digestion was done with complete 1640 medium-(RPMI), containing 20% fetal bovine serum (FBS), 1% nonessential amino acids, and 0.2% penicillin/streptomycin (Invitrogen, Carlsbad, CA, USA). The mixture was filtered and centrifuged at 500 g for 5 min. Following the serial plating, the cells were cultured in complete medium and incubated at 37 °C, in a 5% CO_2_ humidified atmosphere. Chicken fibroblast DF-1 cells were cultured in Dulbecco’s modified Eagle medium (DMEM) (Gibco, Grand Island, NY, USA) supplemented with 10% FBS and 0.2% penicillin/streptomycin (Invitrogen, Carlsbad, CA, USA), then incubated with 5% CO_2_ at 37 °C humidity. DNA plasmids, miRNA mimic, mimic negative control (mimic NC), miRNA inhibitors, inhibitor negative control (inhibitor NC), small interfering RNA (siRNA), and siRNA negative control (siRNA NC) were transiently transfected into cells using Lipofectamine 3000 reagent (Invitrogen, Carlsbad, CA, USA).

### 2.4. RNA Exaction, cDNA Synthesis and Quantitative Real-Time PCR (qRT-PCR)

All RNAs were exacted using Trizol reagent (TaKaRa, Otsu, Japan) according to the manufacturer’s instructions. The quality and concentrations of the RNA samples were detected by 1.5% agarose gel electrophoresis. Total RNA was employed to synthesize cDNA, using a Primescript RT Reagent Kit with gDNA Eraser (Perfect Real Time) (TaKaRa, Otsu, Japan). Synthesized cDNA libraries were diluted with RNase-free water at a ratio of 1:3 before real-time PCR. Relative mRNA expression levels were detected by qRT-PCR using SsoFast Eva Green Supermix (Bio-Rad, Hercules, CA, USA). *GAPDH* was used as an internal control. Reverse transcription for miRNA was conducted using ReverTra Ace qPCR RT Kit (Toyobo, Osaka, Japan). The specific bulge-loop miRNA qRT-PCR primer for miR-30a-3p and U6 were designed by RiboBio (RiboBio, Guangzhou, China). All qRT-PCR reactions were conducted with a CFX96 system (Bio-Rad, Hercules, CA, USA). All reactions were run in triplicates and fold expression changes were calculated using the comparative 2^–ΔΔCt^ method.

### 2.5. Validation of circHIPK3

Based on the NCBI reference sequences of *HIPK3* (NCBI accession number: NM_001199411.1), convergent and divergent primers were designed to validate the existence of circHIPK3. To confirm the cirHIPK3 junction, genomic DNA, and cDNA from CPMs were used for PCR reaction. All PCR products were sequenced by Sangon Biotech Co Ltd. Sequence analysis was conducted using DNASTAR software (DNASTAR 7.1, http://www.dnastar.com). For RNase R treatment, 2 mg of total RNA was incubated 20 min at 37 °C with RNase R (Epicentre Technologies, Madison, WI, USA), and employed to synthesize cDNA for qPCR. For the control group, the same amount of RNA was incubated 20 min at 37 °C and subsequently used to synthesize cDNA.

### 2.6. Plasmids Construction and RNA Oligonucleotides

For the construction of the circHIPK3 over-expression vector, exon 3 of *HIPK3* was amplified using cDNA, produced from CPMs and cloned into a pCD5ciR vector (Geneseed Biotech, Guangzhou, China) between *EcoRI* and *BamHI* restriction sites. The siRNAs to circHIPK3, which especially target the circHIPK3 rather than the linear HIPK3, were designed and synthesized by Geneseed using the sequence shown in Table 1. The gga-miR-30a-3p mimic, mimic NC, the gga-miR-30a-3p inhibitor and inhibitor NC were synthesized by RiboBio (Guangzhou, China). For the construction of pmirGLO Dual-Luciferase reporter vector, wild-type and mutated sequences in the 3′UTR region of *MEF2C* and the partial region of circHIPK3, which include the predicted binding sites of miR-30a-3p, were synthesized and inserted into pmirGLO vectors (Promega, Madison, WI, USA), according to instructions, using *NheI* and *XhoI* restriction sites. The gga-miR-30a sequence was also synthesized and inserted into pmirGLO vectors.

### 2.7. 5-Ethynyl-2′-Deoxyuridine (EdU) Assay

After 48 h of transfection, the treated CPMs and negative control groups in 24-well plates were incubated with 50 μM 5-ethynyl-20-deoxyuridine (RiboBio, Guangzhou, China) for 2 h at 37 °C. After washing twice, the cells were stained with C10310 EdU Apollo. EdU-stained cells were counted using a Leica DMi8 fluorescent microscope (400× magnification) (Leica, Wetzlar, Germany). The ratio of EDU-stained cells to Hoechst 33342-stained cells was calculated and represented the CPM proliferation rate. Detailed protocols were described in the manufacturer′s instruction.

### 2.8. Flow Cytometry of the Cell Cycle

After 48 h of transient transfection with the over-expression plasmid (blank vector) and siRNA (siRNA NC), CPMs were collected from the 12-well plates and kept overnight in 70% ethanol at −20 °C. The cells were then incubated with 50 μg/mL PI (propidium iodide) (Sigma, Louis, MO, USA), 10 μg/mL RNase A (Takara, Otsu, Japan) and 0.2% (*v*/*v*) Triton X-100 (Sigma, Louis, MO, USA) at 4 °C for 30 min. Lastly, cells were detected with a BD AccuriC6 flow cytometer (BD Biosciences, San Jose, CA, USA), and the results were analyzed by FlowJo7.6 software. 

### 2.9. Cell Counting Kit 8 (CCK-8) Assay

CPMs were seeded in a 48-well plate and cultured in complete medium. After transfection, cell proliferation was detected at 12, 24, 36, and 48 h using the TransDetect CCK Kit (TransGen Biotech, Beijing, China), following the manufacturer’s protocol. Cells were added in 25 uL CCK solution to each well and incubated for 2 h at 37 °C in a 5% CO2 cell incubator. Then absorbance of treated and control groups were measured with a Fluorescence/Multi-Detection Microplate Reader (BioTek, Winooski, VT, USA) by optical density at a wavelength of 450 nm.

### 2.10. Immunofluorescence

For immunofluorescence, after transfection, cells in 12-well plates were fixed for 30 min with 4% formaldehyde. Cells were then permeabilized by adding 0.1% Triton X-100 for 5 min and blocked for 30 min with goat serum. Following overnight incubation at 4 °C with anti-MYHC (B103; DHSB, Iowa City, IA, USA; 0.5 μg/mL), Fluorescein (FITC)-conjugated AffiniPure Goat Anti-Mouse IgG (H + L) (Bioworld, Minneapolis, MN, USA; 1:200) was added to the plate and incubated at room temperature for 1 h. Cell nuclei were stained with DAPI (1:50, Beyotime, Shanghai, China) for 5 min. The images were captured with fluorescence microscopy (Leica, Wetzlar, Germany). The area of cells labeled with anti-MYHC was measured using Photoshop software (Adobe Photoshop CC 2018, Adobe, San Jose, CA, USA), and the total myotube area was calculated as a percentage of the total image area covered by myotubes.

### 2.11. Binding Relationship Prediction and Dual-Luciferase Reporter Assay

To predict the relationship between target genes and miR-30a-3p, miRDB (http://mirdb.org/miRDB/) and RNAhybrid (http://bibiserv2.cebitec.uni-bielefeld.de/rnahybrid) were employed. After seeding DF-1 cells in the 96-well plate and culturing for 24 h, four groups (wild type pmirGLO plasmids and mimic as the treatment, mutated pmirGLO plasmids and mimic, wild type pmirGLO plasmids and mimic NC, mutated type pmirGLO plasmids and mimic NC) were set and co-transfected. For the confirmation of the target relationship between circHIPK3 and miR-30a-3p, another method of Dual-Luciferase reporter assay was employed. Three groups (circHIPK3 over-expression plasmid and miR-30a-3p mimic, pCD5ciR and miR-30a-3p mimic, pCD5ciR and mimic NC) were set and co-transfected with a pmirGLO vector containing the miR-30a sequence. After 48 h, Dual-GLO Luciferase Assay System kit (Promega, Madison, WI, USA) was employed to detect luminescent signals of firefly and Renilla Luciferase with a Fluorescence/Multi-Detection Microplate Reader (BioTek, Winooski, VT, USA). Firefly luciferase activities were normalized to Renilla luminescence in each well. Detailed protocols were described in the manufacturer’s instruction.

### 2.12. Western Blotting 

Briefly, cells were lysed in the radio immune precipitation assay (RIPA) buffer (Beyotime, Shanghai, China) containing phenylmethane sulfonyl fluoride (PMSF) protease inhibitor (Beyotime, Shanghai, China). After incubation on ice for 30 min, the samples were centrifuged at 10,000 g for 10 min at 4 °C, and the supernatant was collected. Proteins were separated by SDS-PAGE and blotted onto nitrocellulose membranes (Whatman, Maidstone, UK), then membranes were probed with primary and secondary antibodies. The primary antibodies used were anti-MYHC (1:1000, B103; DHSB, Iowa City, IA, USA), anti-GAPDH (1:1500, AB-P-R 001, Hangzhou Goodhere Biotech, Hangzhou, China), and anti-Tubulin (1:1000, Beyotime, Shanghai, China). The secondary antibodies used were goat anti-rabbit IgG-HRP (1:5000, BA1054, Boster, Wuhan, China) and peroxidase-goat anti-mouse IgG (1:2500, BA1051, Boster, Wuhan, China). Image J software (d1.47, National Institutes of Health, Bethesda, MD, USA) was used to quantify the band intensity.

### 2.13. Statistical Analysis

All results were presented as a mean ± SEM and were subjected to statistical analysis by two-tailed *t*-test. The level of significance was presented as * (*p* < 0.05), ** (*p* < 0.01) and *** (*p* < 0.001).

## 3. Results

### 3.1. circHIPK3 Differentially Expressed during Embryonic Leg Muscle Development

**Figure 1:**
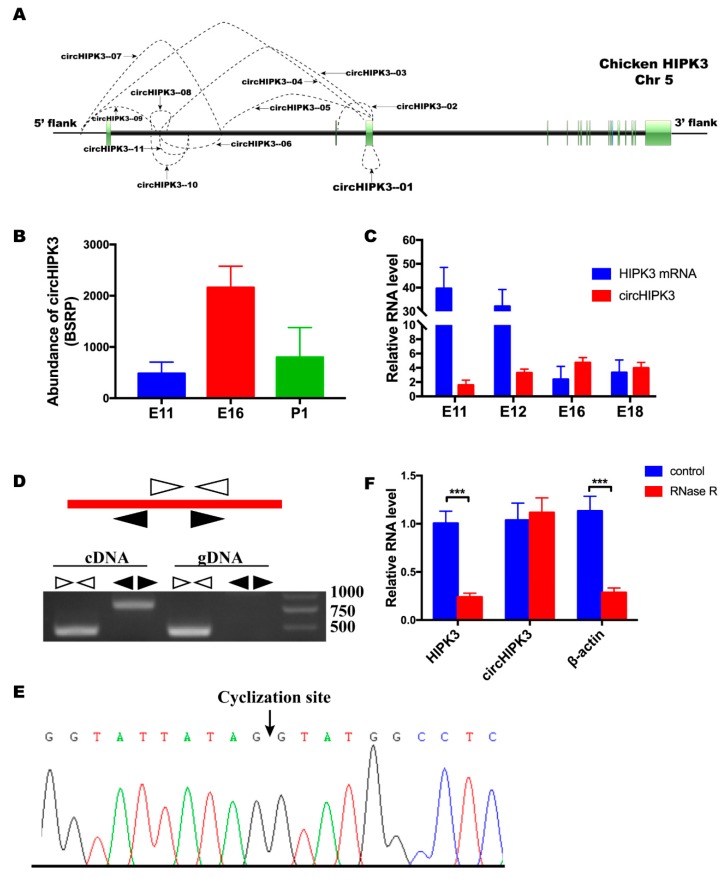
The differential expression and validation of circular HIPK3 (circHIPK3). (**A**) The schema of all circular RNA derived from *HIPK3*. The green rectangles represent the exons of *HIPK3*. (**B**) The RNA-Seq result showed that circHIPK3 was differentially expressed in E11, E16, and P1 of leg muscle. The expressed abundances were normalized as the number of back-spliced reads per million mapped reads (BSRP). (**C**) The expression profiles of circHIPK3 and *HIPK3* mRNA in E11, E12, E16 and E18. (**D**) Divergent primers amplified circHIPK3 in cDNA but not genomic DNA (gDNA). White triangles represent convergent primers and black triangles represent divergent primers. (**E**) Sanger sequencing confirmed the junction sequence of circHIPK3. (**F**) Quantitative real-time PCR (qRT-PCR) showed the resistance of circHIPK3 to RNase R digestion. In all panels, values represent mean ± SEM from three independent experiments. * *p* < 0.05; ** *p* < 0.01; *** *p* < 0.001.

Previous circular RNA sequencing data from our lab revealed 11 circular RNAs were generated by the *HIPK3* gene (available in the Gene Expression Omnibus with accession number GSE89355). The genomic structure of chicken *HIPK3* and the regions, in which all the circular HIPK3 (circHIPK3) RNA were derived, are shown in Figure 1A. Interestingly, circHIPK3 (referred as circHIPK3—01 in Figure 1A), which was derived from exon3 of *HIPK3*, was the only exonic circular RNA. Compared with other circular RNAs derived from *HIPK3*, circHIPK3 had the highest expression level. Its expression level in E16 was significantly higher than in E11 and P1 (Figure 1B). The expression levels of circHIPK3 and *HIPK3* mRNA in E11, E12, E16, and E18 were detected by qRT-PCR (Figure 1C). The trend of the expression level of circHIPK3 was consistent with the result from the sequencing data. However, the expression patterns of circHIPK3 and *HIPK3* mRNA were not identical, which indicated that they might have different functions during leg muscle development. To confirm the sequence and the junction of circHIPK3, genomic DNA and cDNA were used for the PCR reaction, with convergent and divergent primers. The result of the PCR product electrophoresis showed the expectants of convergent primers were amplified with both templates. However, there was no PCR product of divergent primers with the genomic DNA template (Figure 1D). PCR products of divergent primers were analyzed by Sanger sequencing (Figure 1E). Sequencing results showed that circHIPK3 was generated from the third exon of *HIPK3*. The circHIPK3 was also validated by RNase R digestion. The result of qRT-PCR showed that RNase R had no impact on circHIPK3, whereas the levels of linear RNA, HIPK3 and β-actin, were significantly decreased (Figure 1F). These results validated the existence and differential expression of circHIPK3 during skeletal muscle development of chicken.

### 3.2. circHIPK3 Interacts with miR-30a-3p

**Figure 2:**
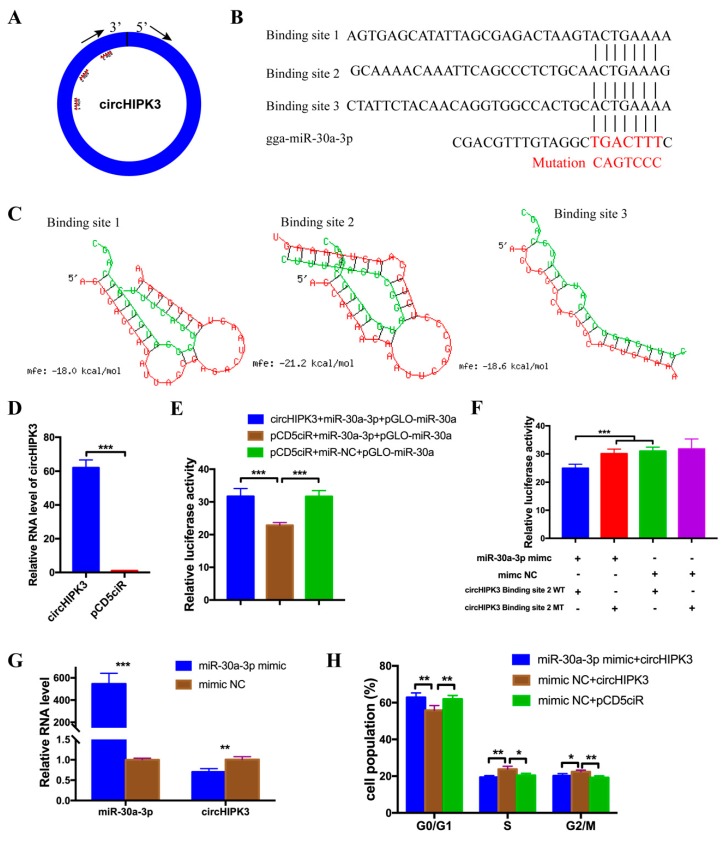
CircHIPK3 interacts with miR-30a-3p. (**A**) A schematic illustration showing the putative binding sites of miR-30a-3p on circHIPK3. (**B**) The potential binding site sequence of miR-30a-3p on circHIPK3. The seed sequences and mutant sequences were highlighted in red. (**C**) The potential interaction model between miR-30a-3p and circHIPK3 from RNAhybrid. (**D**) The expression efficiency of circHIPK3 over-expression vector in DF-1 cells. (**E**) Luminescence was measured after co-transfected with the luciferase reporter and miR-30a-3p mimic (or mimic NC) and circHIPK3 over-expression vector (or pCD5ciR). The relative levels of firefly luminescence normalized to Renilla luminescence are plotted. (n = 6). (**F**) Luminescence was measured after co-transfecting wild type or mutant linear sequence of circHIPK3 with miR-30a-3p mimic (or mimic NC) in DF-1 cells. (n = 6). (**G**) The RNA levels of miR-30a-3p and circHIPK3 from miR-30a-3p mimic transfected DF-1 cells. (**H**) The effect of co-transfected with miR-30a-3p mimic (or mimic NC) and circHIPK3 over-expression vector (or pCD5ciR) on cell-cycle progression of DF-1 cells. The plot of cell-cycle analysis in different cell-cycle phases was compared. In all panels, values represent mean ± SEM from three independent experiments. * *p* < 0.05; ** *p* < 0.01; *** *p* < 0.001.

Many studies showed that circular RNAs exerted their functions by acting as the miRNA sponge. CircHIPK3 was predicted by miRDB and RNAhybrid to be a target of multiple miRNAs. Among these miRNAs, ggs-miR-30a-3p was chosen as a candidate because there were three potential binding sites in circHIPK3 (Figure 2A). The seed sequence of miR-30a-3p matched with three sites in circHIPK3 (Figure 2B). Besides, the prediction results from RNAhybrid indicated that the binding site 2 was the most stable format (Figure 2C). To identify the interactions between circHIPK3 and miR-30a-3p, the over-expression vector of circHIPK3 was constructed and transfected into DF-1 cells. The expression efficiency of the over-expression vector was detected by qPCR. Compared with the group transfected with pCD5ciR, the circHIPK3 over-expression vector expressed a higher level of circHIPK3 (Figure 2D). Then, circHIPK3 over-expression vector and miR-30a-3p mimic were co-transfected into DF-1 cells with a pmirGLO vector, containing the miR-30a sequence. Meanwhile, as the control group, the pCD5ciR and miR-30a-3p mimic (or mimic NC) were co-transfected with the pmirGLO vector, containing the miR-30a sequence. The results showed that the relative luminescence activity of the group with circHIPK3 over-expression vector and miR-30a-3p mimic was significantly higher than the group with pCD5ciR and miR-30a-3p mimic, but had no difference compared with the group pCD5ciR and mimic NC (Figure 2E). These results suggest that circHIPK3 could bind with miR-30a-3p mimic. In addition, sequences which contained binding site 2 or the mutated sequence were inserted into pmirGLO vector. Recombinant vectors with the wild type sequence was then co-transfected into DF-1 cells with miR-30a-3p mimic, meantime, three control groups were set (pmirGLO vector with mutated sequence and miR-30a-3p mimic, pmirGLO vector with wild type sequence and mimic NC, pmirGLO vector with mutated sequence and mimic NC). The results showed that the relative luminescence activity of the group with a wild-type plasmid and mimic was significantly decreased compared to the group transfected with mutated plasmid and mimic, and the group with wild type plasmids and mimic NC (Figure 2F). Moreover, the RNA level of circHIPK3 was significantly down-regulated after over-expression of miR-30a-3p mimic, compared to the group transfected with mimic NC (Figure 2G). Subsequently, the result of flow cytometry analysis showed that miR-30a-3p could reverse the effect of circHIPK3 on a cell cycle (Figure 2H). Altogether, these results indicated that miR-30a-3p could interact with circHIPK3.

### 3.3. miR-30a-3p Inhibits Myoblast Proliferation 

**Figure 3:**
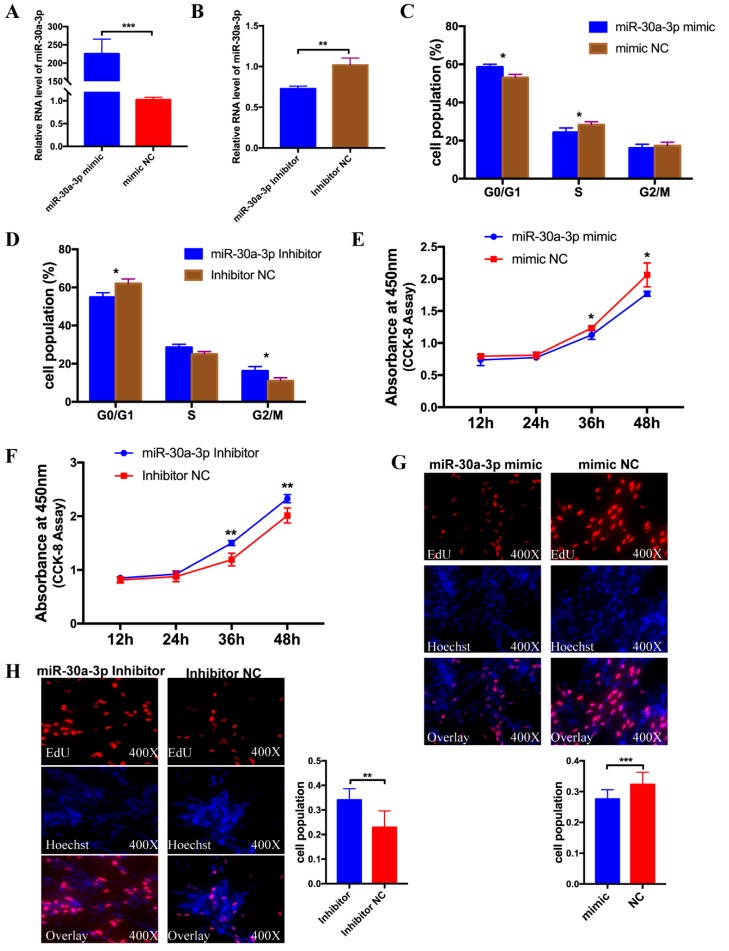
miR-30a-3p inhibits myoblast proliferation. (**A**,**B**) The over-expression and inhibitory effects of miR-30a-3p mimic and inhibitor in CPMs. (**C**,**D**) Effect of miR-30a-3p mimic and inhibitor on cell-cycle progression of chicken primary myoblasts (CPMs). The plot of cell-cycle analysis in different cell-cycle phases was compared. (**E**,**F**) The growth curves of CPMs were measured after the transfection of miR-30a-3p mimic and inhibitor. (**G**,**H**) 5-Ethynyl-2′-Deoxyuridine (EdU) assays for CPMs with over-expression and inhibition of miR-30a-3p. In all panels, values represent mean ± SEM from three independent experiments. * *p* < 0.05; ** *p* < 0.01; *** *p* < 0.001.

To explore the function of miR-30a-3p on the proliferation of CPMs, miR-30a-3p mimic and inhibitor were transfected into CPMs with 100 nM to detect an over-expression effect and an inhibitory effect. The results showed that the two oligos of miR-30a-3p had the effect as expected compared with the mimic NC group, and inhibitor NC group, respectively, and could be used in the subsequent experiments (Figure 3A,B). After being transfected with miR-30a-3p mimic/mimic NC and miR-30a-3p inhibitor/inhibitor NC, flow cytometry analysis was performed in CPMs and the results showed that ectopic expression of miR-30a-3p suppressed the cell cycle markedly, while knock-down of miR-30a-3p significantly promoted the cell cycle (Figure 3C,D). Besides, CCK-8 assay was conducted to detect of proliferation vitality in CPMs. The results showed that the group which transfected with miR-30a-3p mimic had a lower proliferation vitality than mimic NC. In contrast, the group which transfected with miR-30a-3p inhibitor had a higher proliferation vitality than inhibitor NC group (Figure 3E,F). Furthermore, the EdU assay demonstrated that the rate of the cells, which were in the cell division in the ectopic expression group, was significantly less than in the mimic NC group, and the statistics of the cell proliferation rate of the miR-30a-3p over-expression group, were markedly lower than the control group (Figure 3G). Conversely, knock-down of miR-30a-3p dramatically increased the numbers of EdU strained cells compare with the inhibitor NC group (Figure 3H). Altogether, these results indicated that miR-30a-3p could suppress myoblast proliferation.

### 3.4. MEF2C Is a Target Gene of miR-30a-3p

**Figure 4:**
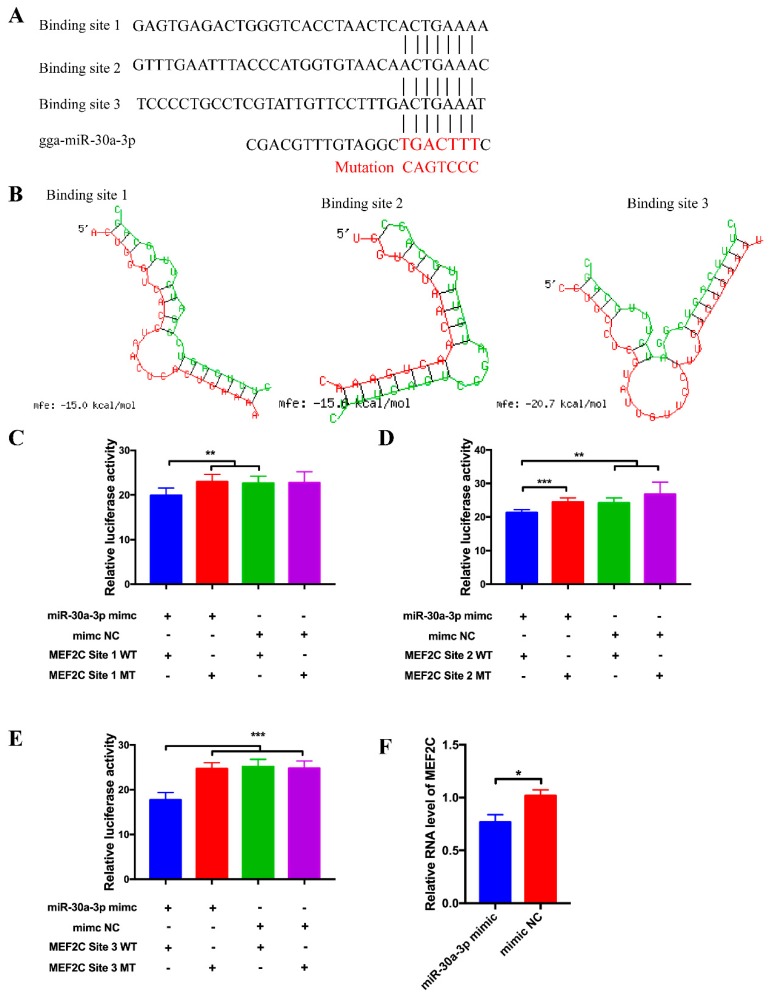
*MEF2C* is a target gene of miR-30a-3p. (**A**) The potential binding site sequence of miR-30a-3p on MEF2C. The seed sequences and mutant sequences were highlighted in red. (**B**) The potential interaction model between miR-30a-3p and MEF2C from RNAhybrid. (**C**–**E**) Luminescence was measured after co-transfecting wild type or mutant sequence of *MEF2C* with miR-30a-3p mimic (or mimic NC) in DF-1 cells. (n = 6). (**F**) The RNA level of *MEF2C* from miR-30a-3p mimic transfected CPMs. In all panels, values represent mean ± SEM from three independent experiments. * *p* < 0.05; ** *p* < 0.01; *** *p* < 0.001.

To investigate the potential function of miR-30a-3p on CPM differentiation, we try to find the differentiation-related genes among the targets of miR-30a-3p. Interestingly, there are three potential binding sites in the 3′UTR region of *MEF2C* (Figure 4A). The potential interaction model from RNAhybrid showed that the binding site 3 was the most stable format (Figure 4B). To validate the target relationship between *MEF2C* and miR-30a-3p, wild-type and mutated-type sequences containing three binding sites, separately, were inserted into the pmirGLO vector for construction of pmirGLO dual-luciferase miRNA target expression vector. After co-transfection of vectors and mimics (or mimic NC), relative luminescence activities were detected by a Fluorescence/Multi-Detection Microplate Reader. For the Binding site 1, the relative luminescence activity of the group, with wild type reporter and mimic, was significantly lower compared with the control groups, which transfected with mutated reporters and mimic, wild type reporter and mimic NC, separately (Figure 4C). For the Binding site 2 and Binding site 3, the luminescence activity of the groups with wild type plasmids and mimic were all dramatically lower than the three control groups (mutated reporters and mimic, wild type reporter and mimic NC, mutated reporters and mimic NC) (Figure 4D,E). Particularly, the binding site 3 exerted the most significant interaction with miR-30a-3p compare with the control groups. The RNA expression level of *MEF2C* was significantly decreased after ectopic expression of miR-30a-3p mimic compared with the group transfected with mimic NC (Figure 4F).

### 3.5. miR-30a-3p Represses CPM Differentiation

**Figure 5:**
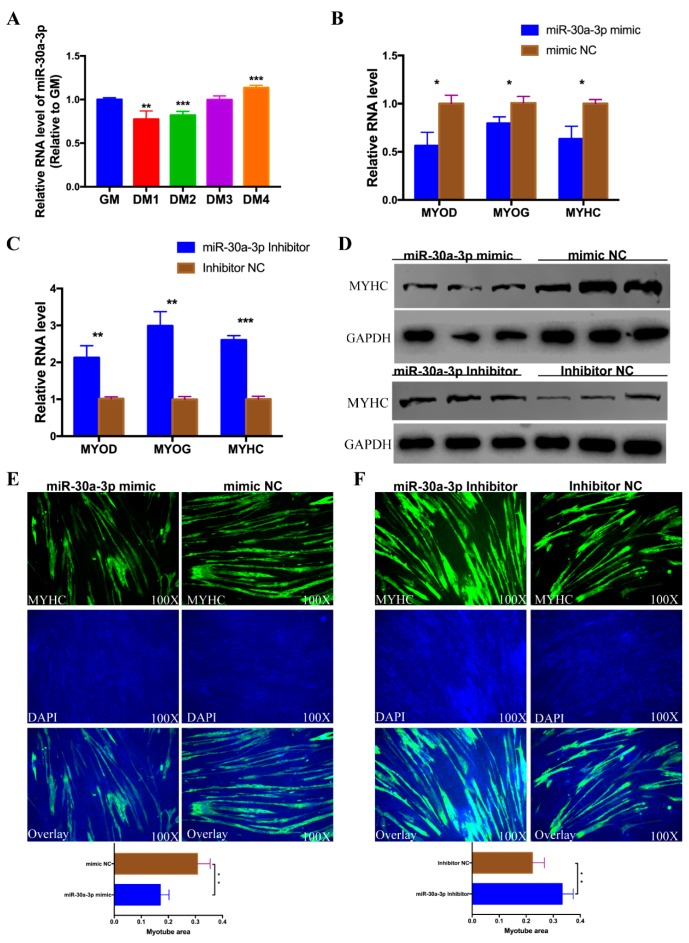
miR-30a-3p represses CPM differentiation. (**A**) The expression profile of miR-30a-3p in the process of CPMs induced differentiation. (**B**) The expression of *MYOD*, *MYOG* and *MYHC* in CPMs after over-expression of miR-30a-3p. (**C**) The expression of *MYOD*, *MYOG* and *MYHC* in CPMs after knock-down of miR-30a-3p. (**D**) The protein level of *MYHC* in CPMs after over-expression and knock-down of miR-30a-3p. (**E**) Immunofluorescence analysis of MYHC-staining cells after over-expression miR-30a-3p in CPMs. (**F**) Immunofluorescence analysis of MYHC-staining cells after knock-down of miR-30a-3p in CPMs. * *p* < 0.05; ** *p* < 0.01; *** *p* < 0.001.

After the confirmation of the target relationship between miR-30a-3p and *MEF2C*, we try to investigate the potential role of miR-30a-3p on CPM differentiation. First, the expression profile of miR-30a-3p was detected in the process of differentiation in CPMs. Interestingly, the expression level of miR-30a-3p was decreased in the first two days of differentiation medium (DM) compare to GM (growth medium), then increased in DM3 and DM4 (Figure 5A). Then, the expression of the myoblast differentiation marker genes, including *MYOD*, *MYOG,* and *MYHC* were evaluated by qPCR in myoblast transfected with miR-30a-3p mimic (or mimic NC) and inhibitor (or inhibitor NC). Over-expression of miR-30a-3p notably inhibited the expression of *MYOD*, *MYOG,* and *MYHC* compared with the groups transfected with mimic NC. Conversely, knock-down of miR-30a-3p promoted the expression of *MYOD*, *MYOG* and *MYHC* relative to the inhibitor NC group (Figure 5B,C). The protein level of *MYHC* was also detected by western blotting. Ectopic expression of miR-30a-3p inhibited the expression *MYHC*, conversely, knock-down of miR-30a-3p promoted the protein expression of *MYHC* (Figure 5D). MYHC immunofluorescence staining was employed on those transfected differentiated myoblasts at DM5. The results showed that the total area of myotubes of miR-30a-3p mimic transfected group was markedly less than that of the group transfected with mimic NC (Figure 5E). On the contrary, the areas of myotubes in miR-30a-3p inhibitor transfected group was more than that of control group (Figure 5F). To sum up, these results revealed that miR-30a-3p could repress CPM differentiation.

### 3.6. circHIPK3 Promotes the Proliferation of CPMs

**Figure 6:**
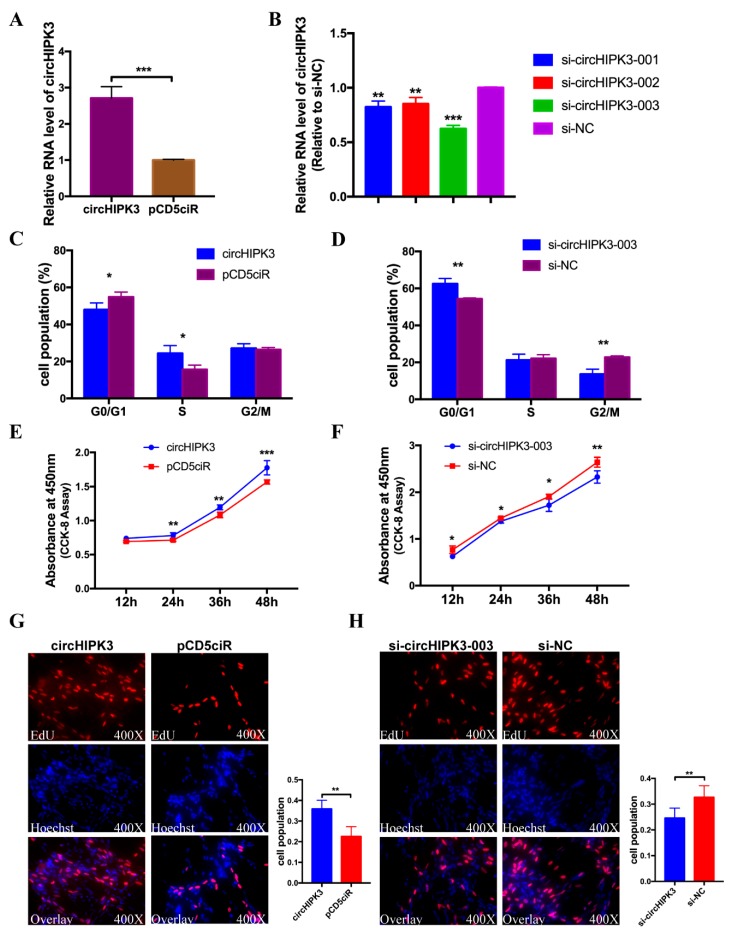
circHIPK3 promotes the proliferation of CPMs. (**A**) The over-expression effect of circHIPK3 over-expression vector in CPMs. (**B**) The interference effects of three siRNAs of circHIPK3 in CPMs. (**C**,**D**) Effect of circHIPK3 on cell-cycle progression of CPMs. The plot of cell-cycle analysis in different cell-cycle phases was compared. (**E**,**F**) The growth curves of CPMs were measured after the transfection of over-expression vector and siRNA of circHIPK3. (**G**,**H**) EdU assays for CPMs with over-expression and inhibition of circHIPK3. In all panels, the values represent mean ± SEM from three independent experiments. * *p* < 0.05; ** *p* < 0.01; *** *p* < 0.001.

Given that circHIPK3 could act as a sponge of miR-30a-3p, and miR-30a-3p could repress the proliferation of CPMs, we hypothesized that circHIPK3 played an opposite role on the proliferation of CPMs. To investigate the role of circHIPK3 on skeletal muscle cell proliferation, the over-expression vector and siRNAs were transfected into CPMs. The over-expression vectors expressed high levels of circHIPK3 compared with the group transfected with pCD5ciR (Figure 6A), and all three siRNAs could significantly knock down the level of circHIPK3 relative to siRNA NC group (Figure 6B). Among the three siRNAs, si-circHIPK3-003 had the highest efficiency of interference effect and was chosen for the following experiments. After being transfected with circHIPK3 over-expression vector/pCD5ciR and si-circHIPK3-003/siRNA NC, flow cytometry analysis in CPMs was performed and the results showed that ectopic expression of circHIPK3 notably promoted the cell cycle, conversely, knock-down of circHIPK3 significantly retarded the cell cycle (Figure 6C,D). Moreover, CCK-8 assay was used to detect the proliferation vitality in CPMs. The results showed that the groups which transfected with circHIPK3 over-expression vectors, had higher proliferation vitality than the negative control group. In contrast, the groups which transfected with si-circHIPK3-003, had lower proliferation vitality than siRNA NC group (Figure 6E,F). The EdU assay indicated that the rate of the cells which were in the cell division in circHIPK3 over-expression group was significantly more than pCD5ciR group, the statistics of the cell proliferation rate of the circHIPK3 over-expression group were markedly higher (Figure 6G). Conversely, knock-down of circHIPK3 decreased the numbers of EdU strained cells dramatically (Figure 6H). In summary, these results demonstrated that circHIPK3 promoted the proliferation of CPMs.

### 3.7. circHIPK3 Promotes the Differentiation of CPMs

**Figure 7:**
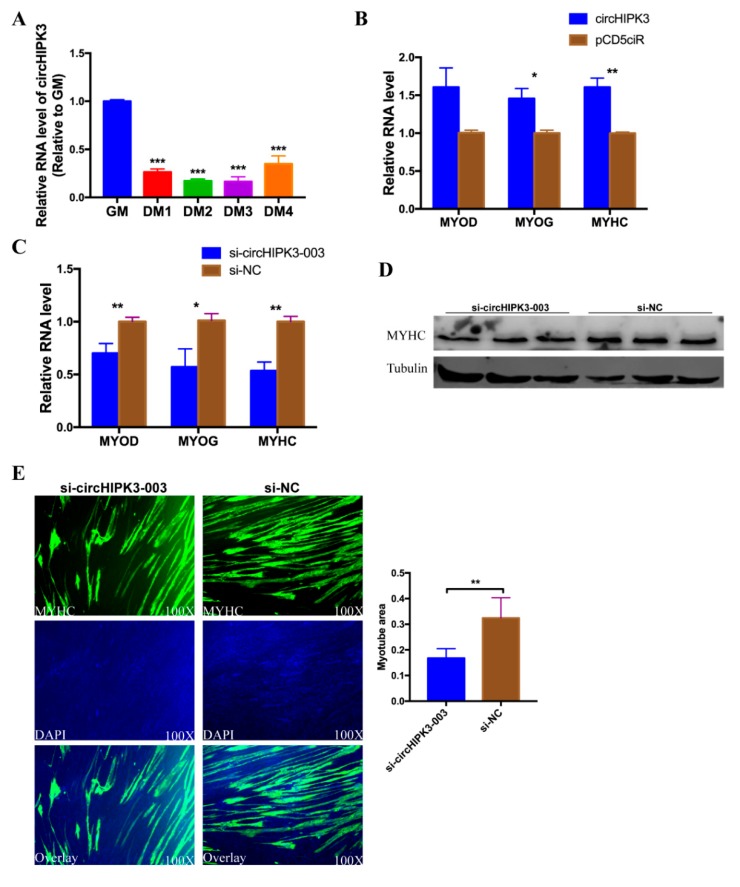
CircHIPK3 promotes the differentiation of CPMs. (**A**) The expression profile of circHIPK3 in the process of CPMs induced differentiation. (**B**) The expression level of *MYOD*, *MYOG* and *MYHC* in CPMs after over-expression of circHIPK3. (**C**) The expression of *MYOD*, *MYOG* and *MYHC* in CPMs after knock-down of circHIPK3. (**D**) The protein level of *MYHC* in CPMs after knock-down of circHIPK3. (**E**) Immunofluorescence analysis of MYHC-staining cells after knock-down of circHIPK3 in CPMs, * *p* < 0.05; ** *p* < 0.01; *** *p* < 0.001.

As the miR-30a-3p had the effect on CPM differentiation, and miR-30a-3p could interact with circHIPK3, we speculated that circHIPK3 might have the opposite effect on CPM differentiation. To confirm our hypothesis, the expression profile of circHIPK3 was detected in the process of differentiation in CPMs. Interestingly, the expression trend of circHIPK3 coincided with miR-30a-3p, and it dramatically decreased in the DM phase compare to GM but raised gradually in DM4 (Figure 7A), indicating that circHIPK3 may be related to the differentiation of CPMs. Then, the expression level of the myoblast differentiation marker genes including *MYOD*, *MYOG* and *MYHC* were evaluated by qPCR in CPMs transfected with circHIPK3 over-expression vector/pCD5ciR and siRNA/siRNA NC. The expression levels of *MYOG* and *MYHC* but not *MYOD* were significantly increased in CPMs transfected with the over-expression vector compare with the group transfected with pCD5ciR (Figure 7B). On the contrary, knock-down of circHIPK3 significantly inhibited the expression of *MYOD*, *MYOG* and *MYHC* compare with the siRNA NC group (Figure 7C). The relative protein level of *MYHC* was also decreased after knock-down of circHIPK3 (Figure 7D). Moreover, MYHC immunofluorescence staining was conducted on transfected differentiated CPMs at DM5. The results showed that the total area of myotubes of si-circHIPK3-003 transfected group was notably less than that of siRNA NC group, and the statistics of the myotube area rate of the si-circHIPK3-003 group were markedly lower than that of siRNA NC group (Figure 7E). These results suggested that circHIPK3 had counteractive effect of miR-30a-3p on CPM differentiation and knock-down of circHIPK3 suppressed the differentiation of CPMs.

## 4. Discussion

Recently, circular RNAs have been identified as a new regulatory factor in multiple biological processes in different kinds of species [18,22,23,24]. Generally, the expression pattern of circular RNAs were corresponding to their linear parental transcript, the expression levels of circular RNAs were lower and had less functions compare to the corresponding mRNA [25,26]. However, some circular RNAs expressed independently high levels and exerted crucial roles in some special cell lines or tissues [14,27]. Many studies proved that circular RNAs were differentially expressed in different muscle developmental stages and played crucial roles in muscle development [28]. Circular RNA sequencing analysis, in different kinds of muscle cells or tissues, among many species, showed that most of the circular RNAs were exonic circular RNAs and differentially expressed during aging [29,30]. Functional circular RNAs in myoblast differentiation play a role by acting as an miRNA sponge or translating micropeptide [15,29,31,32]. Previous circular RNA sequencing data (GSE89355) showed that circHIPK3 was differentially expressed in three stages of chicken muscle development and expressed the highest level, while compared with the other ten circular RNAs, which was produced by chicken *HIPK3*. In this study, we found that circHIPK3 maintained a high expression level in myoblast and decreased sharply from DM1 to DM4, then the expression trend of circHIPK3 increased. This unique expression pattern suggested that circHIPK3 may have an important impact on CPM proliferation and differentiation.

MicroRNAs have been found to be widespread in various cell types or tissues and exerted a regulatory role in biological development [33,34,35]. It exerted important functions in muscle development and was associated with phenotypic changes in skeletal muscle [36,37]. miR-30a-3p is an inhibitor of proliferation in cancer cells [11,12]. In chicken skeletal muscle development, miR-30a-3p differentially expressed in three stages [38]. In this study, we demonstrated that miR-30a-3p inhibited the proliferation of CPMs and suppressed the differentiation by inhibiting the expression of *MEF2C*. According to the resources of microRNA viewer (http://people.csail.mit.edu/akiezun/microRNAviewer/index.html) (Supplementary Figure), miR-30a-3p was conserved in multiple species. The current study provided information to similar studies in other species.

Circular RNA that possesses miRNA response elements (MREs) is known to be a sponge for miRNAs [39]. Previous studies confirmed that the miR-30a family could bind to circHIPK3 in mice [40]. Given the homology of circHIPK3 and miR-30a family, we found that circHIPK3 possessed three potential binding sites for miR-30a-3p, through bioinformatic analyses by miRDB and RNAhybrid. Then two methods were conducted and we confirmed there are interactions between circHIPK3 and miR-30a-3p. Therefore, we hypothesized that circHIPK3 might play an opposite role of miR-30a-3p on skeletal muscle development.

CircHIPK3 was found to be involved in cell proliferation by acting as a sponge of multiple miRNAs, and *HIPK3* gene could produce several circular RNAs with different types in previous study [40,41]. miR-30a inhibited the proliferation by involving in different pathways in different kinds of cells [11,42,43]. In this study, we found that circHIPK3 promoted the proliferation and differentiation of CPMs by combing with miR-30a-3p. However, the underlying mechanism of miR-30a-3p retarding the myoblast proliferation needs further investigation. 

In conclusion, our results suggested that miR-30a-3p could inhibit the proliferation of CPMs and repress the differentiation of CPMs by decreasing the expression of *MEF2C*, while circHIPK3 could promote the proliferation and differentiation of CPMs by sponging miR-30a-3p.

## Figures and Tables

**Table 1:** Primers and RNA oligos used in this study.

Name	Nucleotide Sequences (5′→3′)	Tm. (°C)	Product Size (bp)	Application
QcircHIPK3	F: GTTTAATCCACGCTGACCTCA	61.3	130	qPCR for circHIPK3
	R: GACTTGTGAGGCCATACCTATA			
QHIPK3	F: GGGGTATGTCCCGGAG	61.3	261	qPCR for HIPK3
	R: CTTCGCTAATGGAACAACAC			
QMEF2C	F: AGGGTGTATGTGCAGGAACG	60	288	qPCR for MEF2C
	R: AGCAATCTCGCAGTCACACA			
Convergent primers	F: TGGTACAAGCGGAGATGG R: TTGAGGTCAGCGTGGATTA	55	450	Amplification of partial sequence of exon 3 of HIPK3
Divergent primers	F: GCACGCCAAGGACAAATA	58	782	
	R: TACGCTTCAATCCACATCG			Amplification of partial sequence of circHIPK3 which contain the joint site
β-actin	F: CTCCCCCATGCCATCCTCCGTCTG	52–65	179	qPCR forβ-actin
	R: GCTGTGGCCATCTCCTGCTC			
si-circHIPK3-001	CCCGGTATTATAGGTATGG	-	-	-
si-circHIPK3-002	GGTATTATAGGTATGGCCT	-	-	-
si-circHIPK3-003	ATTATAGGTATGGCCTCAC	-	-	-

Note: The nucleotide sequences of si-circHIPK3 represent the target sequences of each siRNA.

## Supplementary Materials

The following are available online at http://www.mdpi.com/2073-4409/8/2/177/s1.
