# Supplementary figures and images for: Circular RNA circHIPK3 Promotes the Proliferation and Differentiation of Chicken Myoblast Cells by Sponging miR-30a-3p
Source: Cells. 2019 Feb 19;8(2):177. doi: 10.3390/cells8020177 (PMC6406597; doi:10.3390/cells8020177)

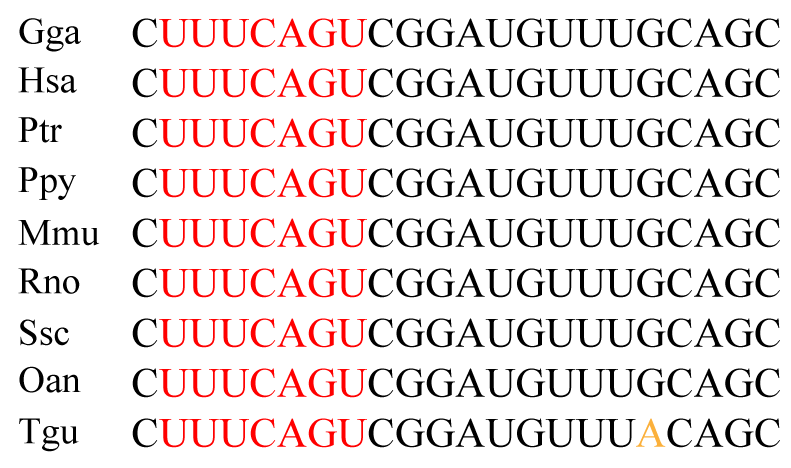

Supplement: Supplementary file 1 [file cells-08-00177-s001.zip › cells-444392-SI.tif]
